# The Interactome of Cancer-Related Lysyl Oxidase and Lysyl Oxidase-Like Proteins

**DOI:** 10.3390/cancers13010071

**Published:** 2020-12-29

**Authors:** Sylvain D. Vallet, Coline Berthollier, Romain Salza, Laurent Muller, Sylvie Ricard-Blum

**Affiliations:** 1 Univ Lyon, University Claude Bernard Lyon 1, CNRS, INSA Lyon, CPE, Institute of Molecular and Supramolecular Chemistry and Biochemistry, UMR 5246, F-69622 Villeurbanne CEDEX, France; sylvain.vallet@univ-lyon1.fr (S.D.V.); coline.berthollier@etu.univ-lyon1.fr (C.B.); romain.salza@gmail.com (R.S.); 2 Center for Interdisciplinary Research in Biology (CIRB), Collège de France, CNRS, INSERM, PSL Research University, 75231 Paris CEDEX 05, France; laurent.muller@college-de-france.fr

**Keywords:** extracellular matrix, interaction networks, lysyl oxidase, lysyl oxidase-like, LOXL2, integrin, Bio-Layer Interferometry, surface plasmon resonance imaging

## Abstract

**Simple Summary:**

The members of the lysyl oxidase (LOX) family initiate covalent cross-linking of the extracellular matrix and contribute to cancer progression. The aim of this study was to build and analyze the first draft of the interaction network of the five members of the LOX family and to determine if it was rewired in cancer. We identified 14 new partners of LOXL2, including α5β1 integrin, and built an interactome of the LOX family comprising 320 proteins, 5 glycosaminoglycans, and 399 interactions. Computational analyses showed that this network participates in extracellular matrix organization and degradation, in cell-matrix interactions, in protein folding and in chaperone activity, and that it is rewired in colorectal carcinoma, with a switch from extracellular matrix organization to protein folding and chaperone activity. This study provides new insights into the molecular mechanisms underlying LOX family involvement in cancer.

**Abstract:**

The members of the lysyl oxidase (LOX) family are amine oxidases, which initiate the covalent cross-linking of the extracellular matrix (ECM), regulate ECM stiffness, and contribute to cancer progression. The aim of this study was to build the first draft of the interactome of the five members of the LOX family in order to determine its molecular functions, the biological and signaling pathways mediating these functions, the biological processes it is involved in, and if and how it is rewired in cancer. In vitro binding assays, based on surface plasmon resonance and bio-layer interferometry, combined with queries of interaction databases and interaction datasets, were used to retrieve interaction data. The interactome was then analyzed using computational tools. We identified 31 new interactions and 14 new partners of LOXL2, including the α5β1 integrin, and built an interactome comprising 320 proteins, 5 glycosaminoglycans, and 399 interactions. This network participates in ECM organization, degradation and cross-linking, cell-ECM interactions mediated by non-integrin and integrin receptors, protein folding and chaperone activity, organ and blood vessel development, cellular response to stress, and signal transduction. We showed that this network is rewired in colorectal carcinoma, leading to a switch from ECM organization to protein folding and chaperone activity.

## 1. Introduction

Lysyl oxidase (LOX) was discovered in 1968 by Pinnell and Martin [1], and its canonical role is to catalyze the first step of the covalent cross-linking of two major extracellular matrix (ECM) proteins, collagens and elastin [2]. Four related-proteins, sharing LOX catalytic domain but diverging at their N-terminus, have been identified in mammals and termed lysyl oxidase-like 1, 2, 3, and 4 (LOXL1, LOXL2, LOXL3, and LOXL4) [3,4,5,6,7]. An N-terminal propeptide exists in LOX and LOXL1, whereas LOXL2-4 have four Scavenger Receptor Cysteine Rich domains (SRCR 1-4) at their N-terminus [2].

All the members of the LOX family have an amine oxidase activity, but they also fulfill biological roles independently of their catalytic activity [8], as shown for example for LOXL2, which is required for initial vessel formation [9] and promotes rhabdomyosarcoma progression [10]. The LOX family plays additional, non-canonical, roles in organ development [11] and cancer [12,13], as shown for LOX [14], LOXL1 [3], LOXL2 [4], LOXL3 [5], and LOXL4 [15,16], and reviewed in [17,18,19,20,21,22,23,24,25]. Beside their activity as amine oxidases, LOXL2 regulates extracellular and intracellular cell signaling pathways [4], LOXL3 is able to deacetylate and deacetyliminate STAT3 to control inflammatory response [26], and LOX and LOXL2 regulate transcription [27]. The enzymes of the LOX family thus exert their biological functions both in the extracellular matrix and within the cells. 

As stated by Tenti and Vanucci [28], “a better understanding of LOXs and their interactions with the different elements of the tumor immune microenvironment will prove invaluable in the design of novel anti-tumor strategies”. It is thus of major importance to identify the partners of the LOX family in order to selectively block interactions for therapeutic purpose, not only for cancers [29] but also for fibrosis. The members of LOX family are therapeutic targets for liver fibrosis indeed [30], mostly LOX, LOXL1, and LOXL2 [31,32]. We have built the interaction network of the N-terminal propeptide of LOX [33] and the protein-protein interaction network of LOXL4 is the only interactome of the LOX family available so far [34]. 

The aim of this study was to build the first draft of the interactome of the five members of the LOX family in order to determine the biological processes it is involved in, identify the major biological-signaling pathways mediating its functions, and if and how it is rewired in cancer. In vitro binding assays, combined to queries of interaction databases and large interaction datasets, were used to retrieve interaction data. We identified 31 new interactions and 14 new partners of LOXL2 and its N-terminal domains, including the α5β1 integrin, involved in both angiogenesis and cancer. This is, to the best of our knowledge, the first report of a direct interaction between a member of the LOX family and an integrin. We built the first draft of the interactome of the LOX family, which comprises 320 proteins, 5 glycosaminoglycans, and 399 interactions. Computational analyses showed that the global LOX family interactome contributes to ECM organization, cell-matrix interactions, protein folding and chaperone activity (with a focus on cytoskeleton proteins), blood vessel development, cellular response to stress, and signal transduction. The partners of LOX, LOXL2, and LOXL3 are enriched in extracellular proteins, whereas LOXL4 partners are enriched in cytosol proteins. The molecular function significantly enriched in the partners of the LOX family members is “Extracellular matrix structural constituent” in contrast to those of LOXL4, which are involved in “Chaperone activity”. We also report that the LOX family has twice more partners identified in tumor cells than in normal cells as shown in the BioPlex 3.0 study [35]. This induces a pathway switch of the LOX family interactome from ECM organization to chaperone activity, a decrease in extracellular or secreted proteins, and in the transcriptional repressor CTCF a zinc finger protein which organizes chromatin, and acts as a transcription factor and a tumor suppressor [36]. The LOX family interactome is thus significantly rewired in cancer cells. The terms “Epithelial-mesenchymal transition” and “Integrin cell surface interactions” are significantly enriched in mesothelioma but not in ovarian cancer, suggesting that the changes in LOX and LOXL interactome associated with cancer might depend on the cancer type. The major goal of our study, which combines both in vitro and in silico computational approaches, was thus to build and analyze the interaction network of LOX and LOXLs to generate new insights and hypothesis on the LOX family functions, and its role in cancer. 

## 2. Results

### 2.1. The Interaction Dataset of the Lysyl Oxidase Family

Several sources of interaction data were combined to build a comprehensive interactome of the lysyl oxidase family. Experimental data were collected by querying manually curated databases MatrixDB [37,38,39,40] and IMEx databases, including IntAct [41,42,43], manually curating additional interactions, querying the large BioPlex 2.0 [44] and 3.0 [35] datasets generated by large-scale affinity purification-mass spectrometry, and performing in vitro binding assays. Surface plasmon resonance imaging (SPRi) binding assays were first performed using the protein and glycosaminoglycan (GAG) arrays we developed [33,45,46] comprised of 44 different biomolecules to identify new extracellular and membrane partners of LOXL2, which plays a prominent role in cancer, and of its N-terminal scavenger receptor cysteine-rich domains (SRCR). LOXL2 was the only member of the LOX family expressed by us and available to us in a sufficient amount to perform in vitro binding assays. Additional, classical, SPR assays were performed to investigate the possible binding of LOXL2 to the ectodomain of collagen XIII, laminin-111, perlecan, and *α*5*β*1 integrin. Furthermore, Bio-Layer Interferometry assays were performed to characterize LOXL2–*α*5*β*1 integrin interaction. Kinetics and affinity parameters were calculated using SPR binding assays performed on dextran-coated sensor chips because the dextran flexibility favors the accessibility of ligand binding sites compared to the bare gold arrays used in SPRi. Furthermore, bare gold arrays usually stabilize the interactions, which might affect the dissociation rate.

#### 2.1.1. Identification of New Partners of LOXL2 by In Vitro Binding Assays

**Figure 1:**
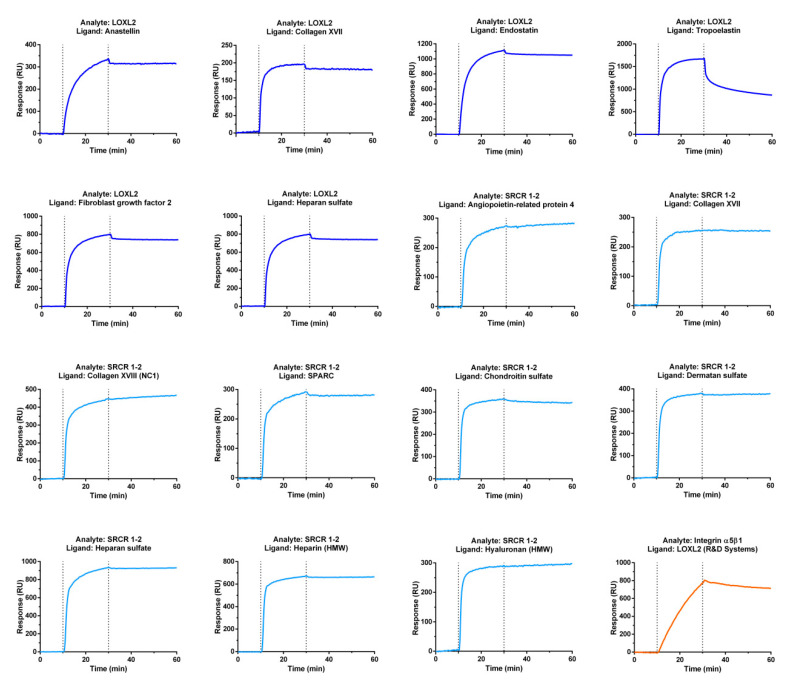
Partners of LOXL2 identified by surface plasmon resonance imaging binding assays. LOXL2 and its N-terminal SRCR domain SRCR 1-2 were injected and recirculated over ECM protein and GAG arrays probed by SPR imaging. Non-specific binding to the array surface was subtracted from the raw signals obtained on protein and GAG spots to get specific binding. The resulting sensorgrams were smoothed. The analytes were color-coded (LOXL2: deep blue, SRCR 1-2: light blue, *α*5*β*1 integrin: orange). Tropoelastin, previously identified by surface plasmon resonance as a binding partner of LOXL2 [50], was used as a positive control. HMW: high molecular weight, dotted lines: start and end of the recirculation phase).

We identified, by SPR imaging and SPR, 14 new partners and 31 new interactions mediated by LOXL2 or its N-terminal domains, SRCR 1-2 and SRCR 1-4. These new, unique partners include five ECM or secreted proteins (angiopoietin-like protein 4, collagen XVIII, fibroblast growth factor-2, laminin-111, and SPARC), three membrane proteins (*α*5*β*1 integrin and the ectodomains of collagens XIII and XVII), one proteoglycan (perlecan) and five GAGs (Figure 1, Supplementary Table S1). LOXL2 bound to sulfated GAGs (chondroitin sulfate, dermatan sulfate, heparan sulfate and heparin) and to hyaluronan, which is not sulfated. LOXL2 interacted with several basement membrane components including perlecan, collagen XVIII via its C-terminal NC1 domain, laminin-111, and SPARC. LOXL2 also bound to ECM and secreted proteins regulating angiogenesis, namely angiopoietin-related protein 4 (ANGPTL4), fibroblast growth factor-2 (FGF2), and two ECM bioactive fragments inhibiting angiogenesis, anastellin and endostatin, cleaved from fibronectin and collagen XVIII, respectively. Last, we showed that LOXL2 interacted with full-length *α*5*β*1 integrin (Figure 1, Supplementary Figure S1, Supplementary Table S1), which also regulates angiogenesis, and with the ectodomain of the transmembrane collagens XIII and XVII (Supplementary Figure S2, Supplementary Table S1), which are located at the surface of keratinocytes [47] and at the neuromuscular junction in the synaptic basement membrane for collagen XIII [48], and at the surface of keratinocytes and in the skin basement membrane zone for collagen XVII [49].

Several LOXL2 partners did not bind to the full-length protein in SPRi assays but only to its SRCR domains as shown for four GAGs, angiopoietin-related protein 4, the NC1 domain of collagen XVIII, and SPARC (Supplementary Table S2, Supplementary Figure S3). The glycosaminoglycans bound to the N-terminus of LOX (the propeptide), and of LOXL2 (the SRCR domains), except for heparan sulfate, which interacted with both the N-terminal SRCR 1-2 and 1-4 domains and full-length LOXL2 (Supplementary Table S2, Supplementary Figure S3). In SPR assays, immobilized SPARC interacted with full-length, soluble, LOXL2, which bound to two non-competitive sites on SPARC, as shown by the fitting of SPR experimental data to an heterogeneous ligand model (Supplementary Figure S2). Both sites on SPARC had similar affinity for full-length LOXL2 (K**_D1_** = 37.8 ± 6.7 nM and K**_D2_** = 41.5 ± 6.9 nM, n = 2, χ^2^ = 0.535 and 0.644). Both complexes had a similar dissociation rate (k_d1_ = 6.22 *×* 10^−3^ and k_d2_ = 7.69 × 10^−3^ s*^−^*^1^), and thus a similar stability and half-life. Their association rates were also similar (k_a1_ = 1.86 × 10^5^ M*^−^*^1^s*^−^*^1^ and k_a2_ = 1.67 × 10^5^ M*^−^*^1^s*^−^*^1^). The SRCR 1-2 domain bound to SPARC according to a two-state model with a higher affinity than full-length LOXL2 (K**_D_** = 21.4 ± 2.5 nM, n = 2, χ^2^ = 1.33 and 2.87). This might be due to the fact that there is a single binding site in the SRCR 1-2. Furthermore, we showed by Bio-Layer Interferometry (BLI) that *α*5*β*1 integrin bound to two sites on immobilized LOXL2 (heterogeneous ligand model, K**_D1_** = 53.8 nM, K**_D2_** = 1.75 µM, k_a1_ = 5.97 × 10^3^ M*^−^*^1^s*^−^*^1^, k_d1_ = 3.21 × 10*^−^*^4^ s^−1^, k_a2_ = 6.31 × 10^3^ M*^−^*^1^s*^−^*^1^, k_d2_ = 1.1 × 10*^−^*^2^ s*^−^*^1^, χ^2^ = 0.0095). The preincubation of *α*5*β*1 integrin with the RGD peptide before dipping the LOXL2-coated sensor in the integrin solution decreased the binding by 24.9%, suggesting that LOXL2 could bind to *α*5*β*1 integrin close to the site recognized by a RGD-containing ligand (Supplementary Figure S1).

#### 2.1.2. The Interactome of the Lysyl Oxidase Family

**Figure 2:**
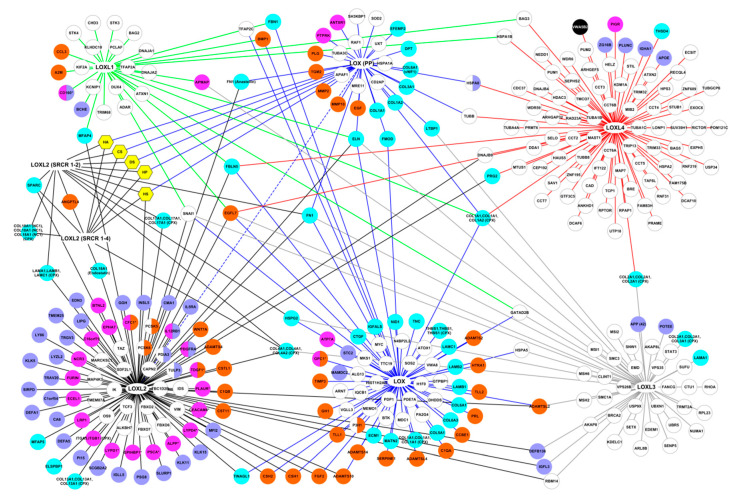
Interactome of the LOX family including the location of their protein binding partners. Proteins represented by nodes were color-coded according to their cellular location(s) determined by the Gene Ontology term “Cellular Component” (Supplementary Table S3): cyan: core-matrisome proteins, orange: matrisome-associated proteins, purple: extracellular proteins not included in the matrisome, white: intracellular proteins, pink: membrane proteins (* glycosyl-phosphatidylinositol-anchored proteins) and black: undetermined location. Yellow hexagons: glycosaminoglycans. Interactions, represented by edges, were color-coded according to the LOX family member they were connected to (blue edges: LOX interactions, green edges: LOXL1 interactions, black edges: LOXL2 interactions, grey edges: LOXL3 interactions, red edges: LOXL4 interactions, dotted blue edges: interactions between two members of the LOX family). The domains containing the binding sites are indicated when known (LOX (PP): lysyl oxidase propeptide, SRCR: Scavenger Receptor Cysteine-Rich).

The complete list of all the interactions retrieved from each source is reported in Supplementary Table S1. The global interactome of the LOX family comprised 320 proteins (including LOX and LOXL 1-4), five glycosaminoglycans and 399 unique interactions (Figure 2). 

LOX and LOXL2 had the highest number of interactions, 107 interactions for LOX, including 36 mediated by its propeptide, which corresponds to 101 unique interactions, and 120 for LOXL2, including 23 mediated by its N-terminal SRCR domains for LOXL2, which corresponds to 103 unique interactions, but they are the most extensively studied members of the family. LOXL1, LOXL3, and LOXL4 established 32, 50, and 91 interactions, respectively (Supplementary Table S1). The high-throughput affinity purification-mass spectrometry (AP-MS) technique used to generate the BioPlex datasets from normal HEK 293 cells and from the tumor cell line HCT 116 allowed the identification of additional interactions of the LOX family. They represented 9.3% of LOX interactions, 25% of LOXL1 interactions, 53.3% of LOXL2 interactions, 36% of LOXL3 interactions, and 82.4% of LOXL4 interactions. However, it should be noted that this approach is based on the purification of protein complexes, which means that the partners of the LOX family identified by this approach did not necessarily bind directly to the LOX family members. These interactions might not be direct, but might represent indirect interactions reflecting proteins identified within a complex containing at least one member of the LOX family.

LOX, its propeptide, and LOXL2 had a high number of partners belonging to the matrisome, whereas the partners of LOXL1, LOXL3, and LOXL4 were mostly intracellular (Figure 2). In addition, membrane proteins, and secreted proteins categorized as extracellular were over-represented in LOXL2 partners compared to the other members of the LOX family. Only one membrane partner was identified for LOXL3 and LOXL4 (adipocyte plasma membrane-associated protein (APMAP), and polymeric immunoglobulin receptor (PIGR), respectively) (Figure 2).

**Figure 3:**
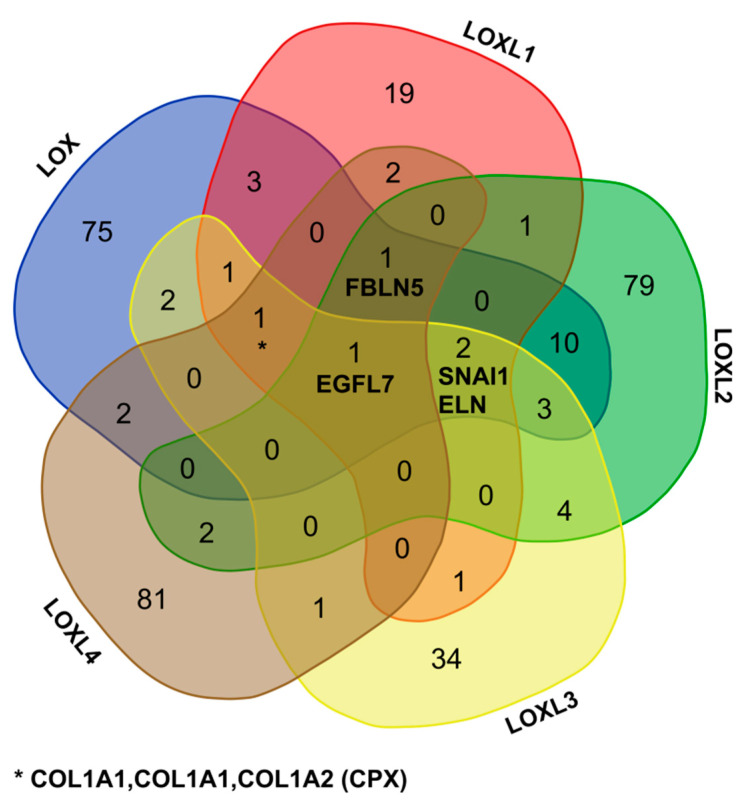
Partners shared by the members of the LOX family. EGFL7: epidermal growth factor-like protein 7, ELN: elastin, FBLN5: fibulin-5, SNAI1: zinc finger protein SNAI1 (Protein snail homolog 1), CPX: complex (e.g., collagen I comprised of two *α*1(I) and one *α*2(I) chains).

The members of the LOX family shared a limited number of partners by pairs, and four members of the LOX family bound to collagen I. Only one protein, the epidermal growth factor-like protein 7 (EGFL7), bound to the five enzymes (Figure 3). Interestingly, we have shown that the propeptide of LOX, which has anti-angiogenic and anti-tumoral activity [51,52], bound to LOXL2 [33], which promotes tumor invasion and metastasis, raising the question of the regulation of angiogenesis and tumorigenesis at the level of the LOX family interactome.

The LOX and LOXL interaction dataset will be freely available via MatrixDB web site (http://matrixdb.univ-lyon1.fr/), the ECM interaction database we have created and we maintain [37,38,39,40], and via the dataset of the International Molecular Exchange consortium (https://www.imexconsortium.org/) to which MatrixDB belongs [41,43].

### 2.2. Analyses of the Interactome of the LOX Family

#### 2.2.1. Enrichment Analysis of the Interactome of LOX Family Using BiNGO

**Figure 4:**
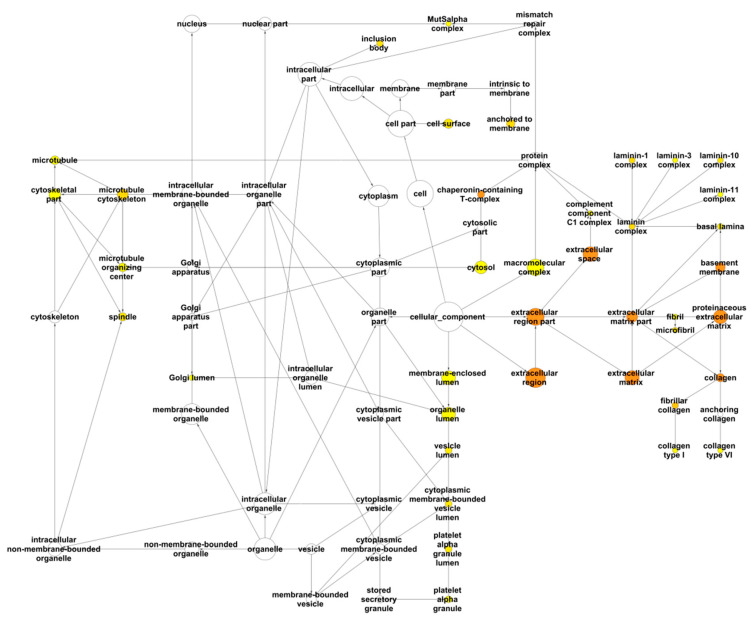
Analysis of the LOX family interactome with BiNGO for the GO term “Cellular Component”. For each GO term category, BiNGO provides a layout of interconnected and colored circles corresponding to each annotation. The circle diameter indicates the number of proteins annotated by a GO term. The yellow to orange color gradient is correlated to the *p*-value (*p* < 0.01), the more significant terms being colored orange.

**Figure 5:**
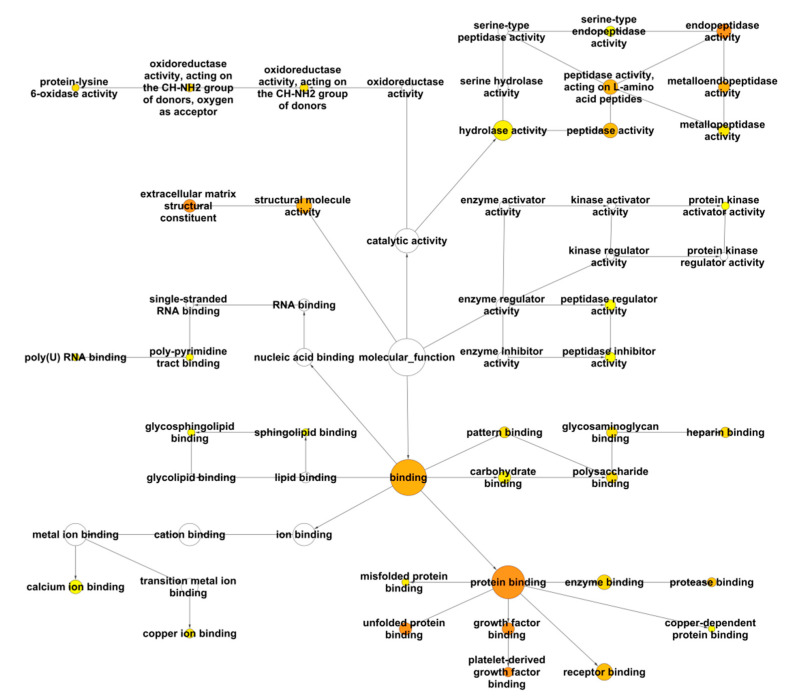
Analysis of the LOX family interactome with BiNGO for the GO term “Molecular Function”. For each GO term category, BiNGO provides a layout of interconnected and colored circles corresponding to each annotation. The circle diameter indicates the number of proteins annotated by a GO term. The yellow to orange color gradient is correlated to the *p*-value (*p* < 0.01), the more significant terms being colored orange.

The complete analysis is available in Supplementary Table S4. The most significantly over-represented annotations for the Gene Ontology (GO) term “Cellular Component” were related to the extracellular matrix and basement membranes, “chaperonin-containing T complex”, “anchored to membrane”, “microtubule cytoskeleton”, and “membrane-enclosed lumen” (Figure 4). Binding, including “Receptor binding” and “Unfolded protein binding”, “Structural molecule activity” (“Extracellular matrix structural constituent”), and “Endopeptidase activity” (“Metalloendopeptidase activity”) were the most over-represented molecular functions in the interaction network of the LOX family (Figure 5). The most significantly over-represented biological processes were “extracellular matrix organization” including “collagen fibril organization”, “response to stress”, “organ development”, including “blood vessel development”, and “macromolecule metabolic process”, including “protein folding” (Supplementary Figure S4).

#### 2.2.2. Enrichment Analysis of the Interactome of LOX Family Using FunRich

The complete analysis is available in Supplementary Table S5. About 41% of the proteins belonging to the LOX family interactome were found in the nucleus, 22.3% in the plasma membrane, 28.7% in exosomes with a significant 2-fold enrichment over the reference proteome, and 38.3% were annotated as extracellular with a 3-fold enrichment compared to the human proteome. The GO term “Extracellular matrix” annotated a limited number of proteins (11%) but it was significantly over-represented in the interactome of the LOX family (13-fold enrichment) compared to the reference proteome (Supplementary Table S5). 50.4% of the network proteins contained a signal peptide, which is consistent with their location in the extracellular space. 

The molecular functions enriched in the interactome of the LOX family were “Extracellular matrix structural constituent”, “Chaperone activity”, “Metallopeptidase activity”, and “Heat shock protein activity”, but these terms annotated a limited number of proteins (≤12%). The over-represented biological processes were “Cell growth and maintenance” (2.7-fold enrichment) and “Protein metabolism” (2.5-fold enrichment) (Supplementary Table S5). The biological pathways over-represented were related to “Integrin cell surface interactions” (7.4-fold enrichment) and chaperonin activity or protein folding, but, although there was a 35-fold enrichment in the pathway, “Formation of tubulin folding intermediates by CCT/TriC”, only 4.5% of the dataset were associated with this pathway (Supplementary Table S5). Last, the zinc finger transcription factor SP1 was over-represented among those regulating the expression of the network proteins.

Enrichment analyses were also performed on the partners of each member of the LOX family (Supplementary Table S6A–E). The extracellular location was enriched in the partners of LOX, LOXL2, and LOXL3 containing 59.1, 58.8, and 37% of extracellular proteins, respectively. Forty percent of LOXL1 partners were extracellular but without a significant enrichment. In contrast, LOXL4 partners were significantly enriched in proteins located in the cytosol (24.7%), the centrosome (18.5%), and the microtubules (11%). The presence of a signal peptide, reflecting secretion outside the cell, was found in ∼70% of LOX (71.1%) and LOXL2 (73.4%) partners, and was significantly enriched in both datasets whereas it was present in 40% of LOXL1 and LOXL3 partners without significant enrichment. The major enriched molecular function associated with LOX (25.8%), LOXL1, LOXL2 (14.4%), and LOX3 was “Extracellular matrix structural constituent”, whereas it was associated with only 4.6% of LOXL4 partners. The major molecular function over-represented in LOXL4 partners was “Chaperone activity”. The major enriched biological process was “Cell growth and maintenance” for LOX and LOXL3 (30.1 and 25% of their partners, respectively, corresponding to a 4.9-fold and a 4-fold enrichment), and “Protein metabolism” for LOXL2 (18.6%) and LOXL4 (19.5%). The major biological pathway enriched in LOX (57.9%, 2.6-fold enrichment), LOXL2 (34.1%, 1.6-fold enrichment), and LOXL3 (46.2%, 2.1-fold enrichment) was “Integrin family cell surface interactions” mediated by β1 or β3 integrin subunits, whereas the major pathways enriched in LOXL4 partners were “Chaperonin-mediated folding”, and those involved in tubulin and actin folding mediated by the molecular chaperone CCT/TriC. It should be noted that most LOXL4 partners were identified by AP-MS performed on cell lysates, which might not be the most appropriate method to identify extracellular protein partners forming insoluble supramolecular assemblies, and might thus favor the identification of intracellular protein partners. The transcription factor CCCTC-binding factor (CTCF), a zinc finger protein was over-represented in LOX partners (36.8%), whereas ZNF513 and ZNF238 were enriched for both LOX and LOXL3 partners (∼20 and 25%, respectively). Last, brain tumor medulloblastoma (23.5%, 39-fold enrichment) was significantly enriched in LOXL3 partners. Furthermore, 21.6% of them were found on the Cancer Gene Census Gene list corresponding to a 8-fold enrichment compared to the human proteome.

#### 2.2.3. Pathway Analysis of the Interactome of LOX Family Using Reactome

**Figure 6:**
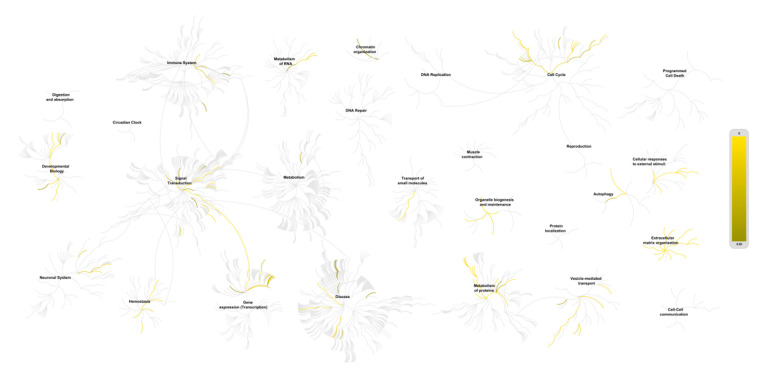
Enrichment analyses of the interactome of the lysyl oxidase family using Reactome. The center of each of the circular “bursts” is the root of one top-level pathway (e.g., “Extracellular matrix organization”, “Metabolism of proteins”, “Cellular Response to external stimuli”, and “Signal transduction”). Over-representation of pathways are color-coded. Pathways which are not significantly over-represented are in light grey. The node size is related to the number of entities inside a pathway.

Reactome is a curated database of pathways and reactions in human biology. Of all the pathways, 973 were hit by a least one protein belonging to the interactome of the LOX family. A genome-wide overview of the pathway analysis of LOX family interactome is displayed in Figure 6. The top-level pathways over-represented in the LOX family interactome compared to the human proteome are “Extracellular matrix organization”, “Metabolism of proteins”, “Cellular Response to external stimuli”, and “Signal transduction”. 

The 100 most relevant pathways identified by Reactome and sorted by *p*-value are listed in Supplementary Table S7. The over-represented sub-pathways in the most over-represented pathway “Extracellular matrix organization” are collagen and elastic fiber formations, assemblies of collagen fibrils and other multimeric structures, and cross-linking of collagen fibrils, as expected, but also extracellular matrix and collagen degradation, “Non-integrin membrane-ECM interactions”, and “Integrin cell surface interactions”. The major sub-pathways over-represented in the “Metabolism of proteins” pathway were “Protein folding”, “Chaperonin-mediated protein folding”, and “Cooperation of Prefoldin and TriC/CCT in actin and tubulin folding”, which reflects the important role played by the network in cytoskeleton organization. Within the “Cellular response to external stimuli” pathway, the most significantly-enriched sub-pathways were “Cellular response to heat stress” and “Regulation of HSF1-mediated heat shock response”. Heat shock factor protein 1 (HSF1) is a stress-inducible and DNA-binding transcription factor, and these pathways highlight the role(s) played by the LOX family within the cell, in addition to their canonical functions in the ECM. The last over-represented pathway among the top 25 pathways is “Signal transduction”, specifically the sub-pathway “MET activates the PTK2 receptor”, which belongs to the sub-pathway “Signaling by receptors tyrosine kinases”. PTK2 is the serine/threonine-protein kinase PTK2/STK2 indeed, and the Met receptor is the hepatocyte growth factor receptor. 

At the level of each LOX family member, “Extracellular matrix organization”, “Degradation of the extracellular matrix”, “ECM proteoglycans”, “Non-integrin membrane-ECM interactions”, and “Integrin cell surface interactions” (Supplementary Table S7) annotations were over-represented, indicating that they are involved in ECM assembly, and in cell-matrix interactions. The common pathways associated with LOX and LOXL3 partners were associated with “Collagen trimerization” and “Assembly of collagen fibrils and other multimeric structures”, whereas the “Elastic fiber formation” pathway was found to be enriched in LOX, LOXL1, and LOXL2 partners. Although its partners were associated with “Extracellular organization” and “Elastin fiber formation” pathways, LOXL1 differed from LOX, LOXL2, and LOXL3 because the most enriched pathways associated with LOXL1 partners are involved in “Cellular response to stress”, and more specifically in “Regulation of HSF-1-mediated heat shock response” (Supplementary Table S7). These pathways were also enriched in LOXL4 partners, but LOXL4 is markedly differed from the other members of the LOX family because the pathways mediating ECM organization, or associated with ECM proteins or proteoglycans, were not enriched in its partners. In contrast, LOXL4 partners were significantly enriched in the protein folding pathway, and specifically in tubulin and actin folding mediated by the molecular chaperone CCT/TriC (Chaperonin containing tailless complex polypeptide 1—CCT—or tailless complex polypeptide 1 ring complex—TriC) (Supplementary Table S7). As noted above, this might be due in part to the fact that numerous partners of LOXL4 were identified by AP-MS.

### 2.3. The Interactome of the LOX Family in Cancer

#### 2.3.1. Comparison of the LOX Family Interactome in HEK 293 and HCT 116 Cells

**Figure 7:**
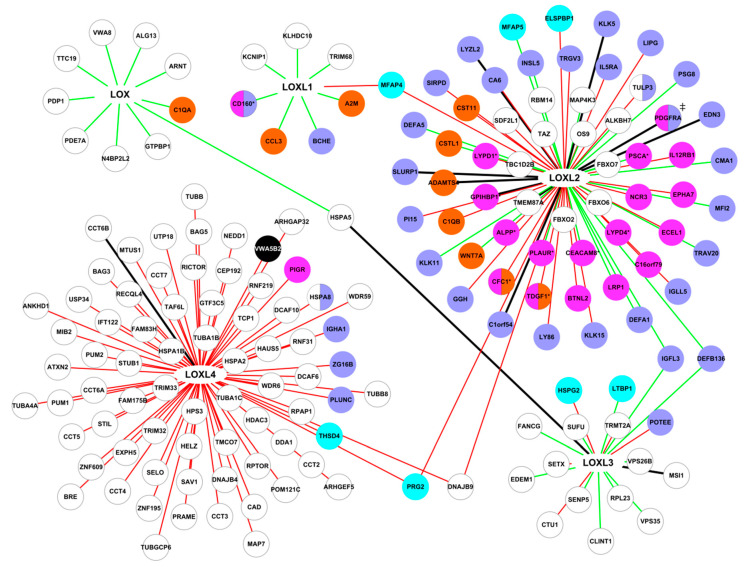
Interactions identified by AP-MS in HEK 293T cells and HCT 116 cells. Green lines: interactions specific to HEK 293 cells, red lines: interactions specific to HCT 116 cells, black lines: interactions found in both cell types. Proteins were color-coded as follows: cyan: core-matrisome proteins, orange: matrisome-associated proteins, purple: extracellular proteins not included in the matrisome, white with grey border: intracellular proteins, pink: membrane proteins, black: undetermined location. ‡: two different isoforms in normal and tumor cells. *: glycosyl-phosphatidylinositol-anchored proteins.

We used the interaction data collected by affinity chromatography-mass spectrometry in normal HEK 293 cells and in the human colorectal carcinoma cell line HCT 116 (BioPlex 3.0 dataset) to compare the interactome of the LOX family in normal and tumor cells. The interaction repertoire of the LOX family differed between these two cell types (Figure 7). The LOX family had twice as many interactions in tumor cells than in normal cells. 63 interactions were established by the LOX family in HEK 293 cells, and 124 in HCT 116 cells. It should be noted that no interaction involving LOX was identified in the tumor cell line HCT 116 in the experimental conditions used (Figure 7). 12 interactions were found in both cell types, LOXL2 being involved in 9 of them, LOXL3 in 2, and LOXL4 in 1. These interactions involved 6 secreted proteins, 4 intracellular proteins, and 2 membrane proteins (Figure 7, Supplementary Table S1). It should be noted that one interaction involved two different isoforms of the platelet-derived growth factor receptor alpha (PDGFRA) in the normal and tumor cells, a membrane form in HEK 293 cells and a secreted form in HCT 116 cells. 51 interactions, and 112 interactions were specific to HEK 293 cells and to the tumor cell line HCT 116, respectively (Supplementary Table S1).

Enriched annotations generated by the FunRich tool for the LOX family partners specific to HEK 293 and those specific to HCT 116 cells were compared. A marked decrease in “Extracellular” location (36.8% in HEK cells versus 21.3% in HCT 116 cells), and a moderate increase in “Cytoplasm” and “Nucleus” were observed for Cellular Component in partners specific to tumor cells, associated with a 2-fold decrease in “signal peptide” (62.5% in HEK293 cells versus 31.5% in HCT 116 cells) (Supplementary Table S8A,B) meaning that the secreted LOX and LOXL protein partners were less abundant in HCT 116 cells. Additional changes in the tumor cell line were a 4-fold increase in “Chaperone activity” (2.1% in HEK cells versus 8.9% in HCT 116 cells), a slight decrease in “Cell communication”, “Signal transduction”, and “Glypican pathway”, and a 3-fold decrease in the transcriptional repressor CTCF (33.3% in HEK cells versus 11.62% in HCT cells) (Supplementary Table S8A,B), a zinc finger protein which organizes chromatin, and acts as a transcription factor and a tumor suppressor [36]. 

The most over-represented biological pathways identified by Reactome in HEK 293 cells were “Molecules associated with elastic fibers”, “Aryl hydrocarbon receptor signaling” and “Elastic fiber formation”, and “Alpha-defensins” (Supplementary Table S9), whereas “Formation of tubulin folding intermediates by CCT/TriC”, “Cooperation of Prefoldin and TriC/CCT in actin and tubulin folding”, “Prefoldin mediated transfer of substrate to CCT/TriC”, “Folding of actin by CCT/TriC”, and “Chaperonin-mediated protein folding” were the most over-represented in tumor cells (Supplementary Table S9), reflecting the role of the LOX and LOXL partners of LOX in chaperonin activities and cytoskeleton formation into tumor cells.

**Figure 8:**
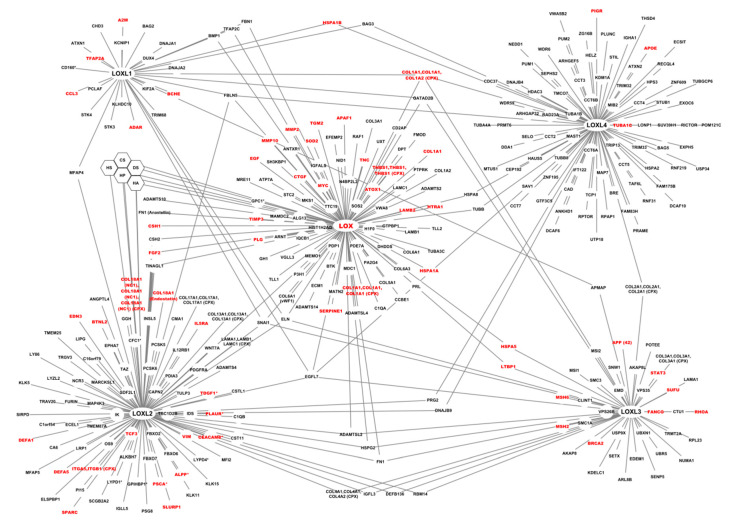
Interactome of the five members of the LOX family. Black: protein partners of LOX and LOXLs, Red: protein partners of LOX and LOXLs (including LOX) annotated as “Cancer-related condition” in Reactome FIViz as described in the material and methods section. Hexagons: glycosaminoglycans, *: glycosyl-phosphatidylinositol-anchored proteins.

Reactome FIViz was used to identify proteins involved in cancer within the complete network. Of the LOX family interactome, 17.5%, corresponding to 56 genes, including the LOX gene, belong to the “Cancer-related condition” class defined in Reactome FIViz (Figure 8). Here, the interactions mediated by the propeptide of LOX and by the SRCR 1-2 and 1-4 domains of LOXL2 were not distinguished from those of LOX and LOXL2, respectively. Moreover, using the functional enrichment tool FunRich and the Catalogue Of Somatic Mutations In Cancer (COSMIC) data, the network of the LOX family was shown to be associated with endometrium (78.9% of the network proteins), prostate (62.3%), ovary (74.7%), and skin (71.1%) cancers (Supplementary Table S5).

**Figure 9:**
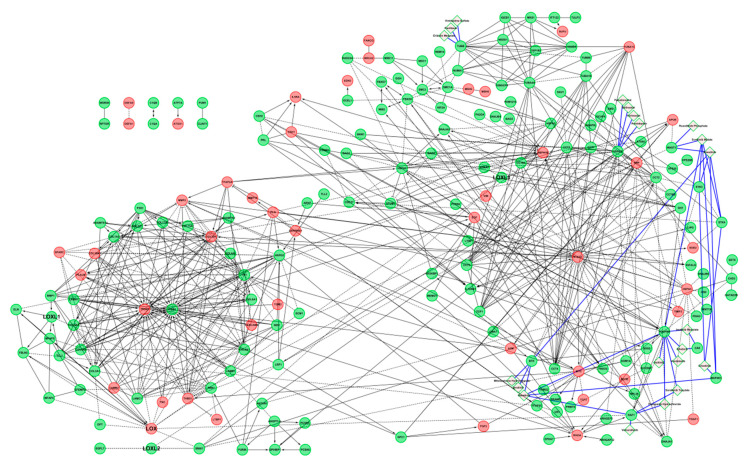
Drug–target interactions identified in the functional interaction network generated using Reactome FIViz. Green: proteins, Red: proteins annotated with cancer-related condition, Diamonds: drugs. Blue edges: interactions between drugs and targets, arrowheads: activating and catalyzing or expression regulation, dotted lines: predicted functional interactions, ⊥ for inhibition, solid line for complexes or reactions.

FDA-approved cancer drugs were visualized in the context of the functional interaction network of the LOX family built by Reactome FIViz, to investigate the potential functional impacts of displayed cancer drugs. However, 128 protein partners of LOX and LOXLs were not functionally linked to any member of the network or any drug, and were not displayed, although 11 of them were associated with cancer. This is due to the lack in Reactome FIViz of functional links for these proteins. EGFL7, for instance, is connected to a single member of the LOX family, although we report here that this protein binds to the five members of the family. The interactome generated in this study will thus be useful to update applications using interaction datasets. Neither LOX, nor LOXLs, were directly associated with an anti-cancer drug. Only eight members of the functional network were connected to anti-cancer drugs, including five tyrosine kinase receptors (MAST1 or Microtubule-associated serine/threonine-protein kinase 1, PDGFRA or Platelet-derived growth factor receptor alpha, BTK or Tyrosine-protein kinase, RAF1 or RAF proto-oncogene serine/threonine-protein kinase, and MAP4K3 or Mitogen-activated protein kinase kinase kinase kinase 3), the tubulin beta chain (TUBB), the histone deacetylase 3 (HDAC3), and carbonic anhydrase 6 (CA6) (Figure 9). 

#### 2.3.2. The Matrisome Part of LOX Family Interactome in Cancer

**Figure 10:**
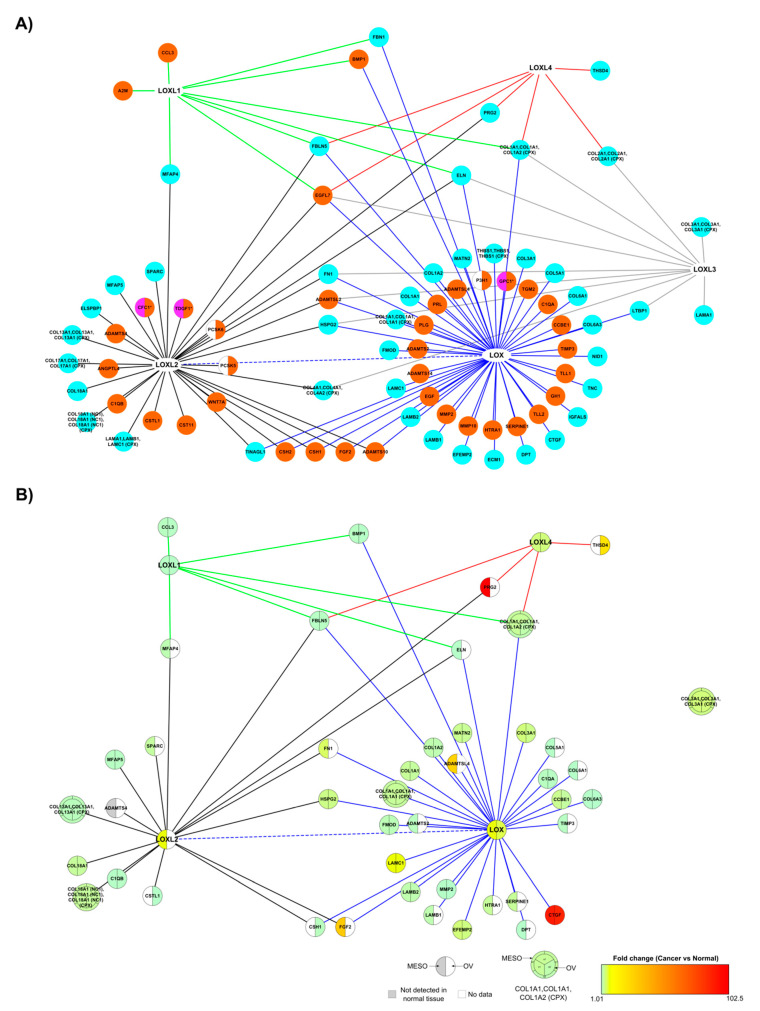
Matrisome-specific interactome of the LOX family. (**A**) Global matrisome-specific interactome of the LOX family. Proteins represented by nodes were color-coded according to their cellular location(s) determined by the Gene Ontology term “Cellular Component” (Supplementary Table S3): cyan: core-matrisome proteins, orange: matrisome-associated proteins, pink: membrane proteins. (**B**) Matrisome-specific interactome of the LOX family in mesothelioma (MESO) and ovarian serous cystadenocarcinoma (OV). Nodes were color-coded using the fold change values calculated in cancer (MESO and OV) versus normal patients in Izzi et al. [53]. Interactions, represented by edges, were color-coded according to the LOX family member they were connected to (blue edges: LOX interactions, green edges: LOXL1 interactions, black edges: LOXL2 interactions, grey edges: LOXL3 interactions, red edges: LOXL4 interactions, dotted blue edges: interactions between two members of the LOX family). Not detected: the expression level of ADAMTS4 was 0 in normal tissue, the fold-change was thus not calculated. No data: the gene is absent from Izzi et al. dataset [53].

We investigated the expression data of matrisome proteins collected by Izzi et al. in 32 tumor types [53] (Supplementary Table S10), to identify those where the members of the LOX family were present. No dataset contained the five members of the LOX family. However, four (LOX, LOXL1, LOXL2, LOXL4) and three (LOX, LOXL1, LOXL4) members were present in the mesothelioma (MESO; 87 patients, 239 genes) and ovarian serous cystadenocarcinoma (OV; 427 patients, 209 genes) datasets. Moreover, the highest fold change in the expression of LOX family members compared to normal tissues were found in MESO and OV patients for LOX (MESO: 7.32, OV: 5.89), LOXL2 (MESO: 7.41), and LOXL4 (MESO: 4.17, OV: 4.17). We, thus, focused on mesothelioma and ovarian datasets to investigate the possible role of the LOX family in cancer. We first built the matrisome-specific interactome of the LOX family, based on the annotations of Supplementary Table S3, and comprised of 86 proteins and 113 interactions (Figure 10A). The matrisome interactome of the LOX family specific to mesothelioma and ovarian cancer was then built by selecting the partners expressed in mesothelioma and in ovarian cancer, and ranking them according to their fold-change in cancer relative to normal tissue (Figure 10B). Of the global LOX network, 42 and 27 proteins were found in the MESO and OV datasets, respectively, with 24 proteins found in both cancer datasets (Supplementary Table S10). Among the common partners, bone marrow proteoglycan or proteoglycan 2 (PRG2) and the matricellular protein connective tissue growth factor (CTGF) exhibited the highest fold changes in the MESO dataset (102.42 and 90.35, respectively), whereas CTGF was also highly overexpressed in the OV dataset (fold change: 87.46). CTGF, a transcriptional target of TGF-β signaling, is a therapeutic target in high grade serous ovarian cancer [54]. All the proteins of the matrisome-specific interactome of the LOX family expressed in ovarian cancer were also expressed in mesothelioma, except cystatin-like 1 (CSTL1), THSD4 (ADAMTSL-6), and chorionic somatomammotropin hormone 1 (CSH1), which were expressed only in ovarian cancer (Figure 10B).

Differences were observed in the matrisome subnetwork of the LOX family between mesothelioma and ovarian cancer. “Epithelial-mesenchymal transition” (37% of proteins annotated, 12.6-fold-enrichment) and “Integrin cell surface interactions” (22%, 19.4-fold-enrichment) were the most significantly enriched pathways in the mesothelioma subnetwork (Supplementary Table S11A) but not in the ovarian cancer subnetwork (Supplementary Table S11B), which suggests that the changes in LOX and LOXL interactome associated with cancer might depend on the cancer type. 

## 3. Discussion

The interactome of the LOX family contains a significant amount of intracellular and membrane proteins in addition to secreted and ECM proteins. Given that some activities of LOX and LOXLs are independent of their enzymatic activities, LOX and LOXLs might qualify as moonlighting proteins. The interactome reported here is enriched in LOXL4 partners (91) compared to the previously reported LOXL4 interactome, which contained 15 partners [34]. To further increase the coverage of the LOX family interactome, ECM calcium-binding proteins could be assayed for their binding to LOX and LOXLs because a calcium-binding site has been found in the 3D structure of a precursor of human LOXL2 [55], and we have shown that this site is conserved in the 3D model of human LOX [56]. The 2-fold enrichment in exosomes in the LOX family interactome suggests that ECM cross-linking by hypoxic endothelial cell-derived exosomes mediated by LOXL2 [57], and exosome-mediated secretion of LOXL4 promoting hepatocellular carcinoma cell invasion and metastasis [15] might involve other members of the family and their partners. The three-dimensional model of LOX we have recently built [56], and the crystal structure of a precursor state of LOXL2 [55], could be used for docking experiments to map the binding sites of their partners, and predict those which could be either substrates or modulators of LOX and LOXL enzyme activity by binding to their catalytic sites. These potential substrates will then be tested in vitro to determine if they are modified by LOX and LOXL2, or if they regulate their enzymatic activity. 

The major processes associated with the LOX family interactome are ECM organization, degradation and cross-linking, protein folding and chaperone activity, organ development, and, in particular, blood vessel development. The over-representation of the “Non-integrin membrane-ECM interactions” and “Integrin cell surface interactions” sub-pathways in the current version of the interactome will prompt us to perform binding assays of LOX and LOXLs with various integrins (we show here that LOXL2 binds to α5β1 integrin), and with other ECM receptors such as discoidin-domain receptors 1 and 2, membrane collagens XXIII and XXV—we report here that LOXL2 interacts with the ectodomain of two other membranes collagens XIII and XVII—and with cell surface proteoglycans such as syndecans and glypicans, inasmuch as the extracellular domain of syndecan-4 is able to promote LOX-dependent cross-linking in a pressure-overloaded heart [58]. Another interesting feature of the LOX family interactome is the over-representation in the network of the transcription factor SP1, which is over-expressed in many cancers [59] in keeping with the role played by LOX and LOXLs in these diseases. The number of partners of LOX family members is doubled in the tumor cell line HCT 116 compared to normal cells, which is consistent with a previous report showing that cancer-associated proteins have on average twice more partners than non-cancer proteins [60]. The rewiring of the interactome in cancer cells is associated with an increase in chaperone activity, a decrease in extracellular and secreted proteins, and in the transcriptional repressor CTCF a zinc finger protein which organizes chromatin, and acts as a transcription factor and a tumor suppressor [36]. The LOX family interactome is thus significantly rewired in colorectal cancer cells, which induces a switch of its major pathway from ECM organization and assembly to chaperone activity and protein folding. The two most relevant pathways sorted by *p*-value found in tumor cells are “Formation of tubulin folding intermediates by CCT/TriC”, and “Cooperation of Prefoldin and TriC/CCT in actin and tubulin folding”. This is consistent with the higher expression of CCT/TRiC in cancer cells than that in normal cells, and with the role of CCT/TRiC-mediated protein folding in maintaining cellular proteostasis and in cancer cell development [61]. 

LOXL2 binds to basement membrane proteins, one proteoglycan, and to several regulators of angiogenesis, which is consistent with previous findings reporting that LOXL2 interacts with collagen IV, a major component of the basement membrane [62]. We proposed that the SRCR domains of LOXL2 orchestrate scaffolding of the vascular basement membrane and angiogenesis through interactions with collagen IV and fibronectin [62]. The interaction of the SRCR domains of LOXL2 with SPARC and the C-terminus of collagen XVIII, both located in basement membranes, could also contribute to the scaffolding of the vascular basement membrane and angiogenesis. Several proteins and GAGs bind to the N-terminal domain of LOX [33] and LOXL2 [62], supporting the fact that LOX family members could exert biological functions independently of their catalytic domain. This has been shown for the propeptide of LOX and LOXL1, which play a role in the formation of elastic fibers via their binding to tropoelastin [63] and for the propeptide of LOX, which has anti-tumoral and anti-angiogenic properties [51,52].

Collagens XIII and XVII, identified in this study as new partners of LOXL2, contribute to anchor keratinocytes to the skin basement membrane [64] in agreement with the role suggested for LOXL2 in the epidermis rather than in dermis [65]. Furthermore, collagens XIII and XVII are both associated with cancer [66,67], and their interactions with LOXL2 could also occur in this context. The binding of LOXL2 to glycosaminoglycans could contribute to ECM assembly, and to its binding to the cell surface. Although there is an interplay between LOX and cell-surface integrin receptors in the tumor microenvironment [68], and between LOX and integrin-mediated mechano-transduction [69], no direct interaction of a member of the LOX family with an integrin has been described so far. This study is the first report of a direct binding of a member of the LOX family to an integrin. The sequence of LOXL2 does not contain the canonical RGD sequence recognized by RGD-dependent integrins such as α5β1 integrin, but it contains a NGR sequence located in the C-terminal part (residues 615-617). It has been shown that the isoDGR motif, generated by asparagine deamidation of a NGR sequence in fibronectin, is an αvβ3 integrin-binding motif, which regulates endothelial cell adhesion and proliferation [70]. The NGR sequence found in LOXL2 could thus participate in integrin recognition. In addition, a conformational RGD motif could be formed in folded LOXL2, but other binding sites are likely present on the integrin because the RGD peptide inhibits only partially the LOXL2-α5β1 interaction. The interaction of LOXL2 with α5β1 integrin warrants further investigation in the context of angiogenesis and cancer because α5β1 integrin is expressed by neovessels and tumor cells [71]. Some effects of LOXL2 on tumor cells might thus be mediated by this integrin. 

## 4. Materials and Methods

### 4.1. Surface Plasmon Resonance (SPR) Binding Assays 

SPR imaging experiments were carried out in a BIAcore Flexchip system (GE Healthcare, Vélizy-Villacoublay, France) using the protein and glycosaminoglycan arrays we have previously developed [33,45,46]. The BIAcore Flexchip is able to monitor simultaneously up to 400 interactions between biomolecules spotted on gold arrays (physically adsorbed on the gold surface) and a protein (the analyte) injected in buffer flow and recirculated over the array. We used this system to identify new partners of LOXL2 and its SRCR domains 1-2 (residues 58-302) and 1-4 (residues 58-544), expressed as recombinant proteins as previously described [62], using 44 unique proteins, five protein fragments, and five glycosaminoglycans from commercial sources or expressed under a recombinant form in the laboratory (Supplementary Table S12). These biomolecules were spotted onto the gold surface of a Gold Affinity chip (GE Healthcare) in triplicate using a non-contact PiezoArray spotter as previously described [33,46]. The regions of interest had four associated reference spots to evaluate non-specific binding of the analyte to the chips. The chips were blocked with a buffer containing mammalian proteins (BR-1007-08, GE-Healthcare) for 5 × 5 min. LOXL2, SRCR 1-2 and SRCR 1-4 domains (500 nM) were recirculated over the arrays for 20 min, and the complexes were dissociated in buffer flow. Only interactions identified in two separate experiments were included in the interactome. α5β1 integrin (29.3 µg/mL) was used as analyte, and recirculated over ECM arrays including spotted LOXL2 in 10 mM Hepes pH 7.4 containing 150 mM NaCl, 50 mM β-octyl-D-glucopyranoside, 2 mM MgCl_2_, and 2 mM MnCl_2_. 

The binding of soluble LOXL2 to the ectodomain of human collagen XVII (Supplementary Table S1, Supplementary Figure S2) was characterized by SPR binding assays performed in a BIAcore T100 system (GE Healthcare, Facility of UMS 3444 Gerland, Lyon, France) as previously described for the characterization of LOXL2-collagen IV interaction [62]. The ectodomain of collagen XVII, fibronectin, laminin-111, and perlecan (HSPG2) were covalently immobilized on a CM5 or a CM3 (collagen XVII) sensor chip using the amine coupling kit (GE Healthcare) according to the manufacturer’s instructions. A control flow cell was prepared by omitting ligands to evaluate the non-specific binding of full-length LOXL2 and its scavenger receptor-cysteine-rich domains (SRCR 1-2 and SRCR 1-4) to the sensor chip surface. LOXL2, SRCR 1-2, and SRCR 1-4 domains diluted in 10 mM Hepes pH 7.4 containing 150 mM NaCl and 0.05% P20 as running buffer were injected over immobilized ligands. LOXL2 was covalently immobilized on a CM5 sensor chip via its primary amino groups, and the ectodomain of human collagen XIII was injected at different concentrations at 30 μL/min for 180 s over immobilized LOXL2 and the control flow cell. The association (ka) and dissociation rate (kd) constants and the equilibrium dissociation constant (K_D_) were calculated using the BIAevaluation software (version 2.0.3). 

### 4.2. Bio-Layer Interferometry (BLI) Binding Assays

The binding of LOXL2 to α5β1 integrin was identified by Bio-Layer Interferometry, a label-free technique monitoring biomolecular interactions in real time, using an Octet RED96 system (FortéBio, Sartorius, Dourdan, France), as previously described [33,72]. Recombinant human LOXL2 (R&D Systems, BioTechne, Noyal Châtillon sur Seiche, France, 2639-AO) was covalently immobilized via its primary amine groups on AR2G biosensors (FortéBio, Sartorius) at 25 ug/mL in sodium acetate buffer 10 mM pH 4. The sensors coated with LOXL2 and control sensors were dipped in human α5β1 integrin (Millipore, Merck, Molsheim, France, CC1027) diluted in 10 mM Hepes pH 7.4 containing 150 mM NaCl, 50 mM β-octyl-D-glucopyranoside, 1 mM MgCl_2_, and 1 mM MnCl_2_. Inhibition experiments were performed by preincubating the α5β1 integrin at 35.5 µg/mL for one hour at room temperature with or without the RGD peptide diluted at 50 µg/mL in the above buffer (Sigma–Aldrich, Saint-Quentin-Fallavier, France, A8052). Kinetics and affinity parameters were calculated using the evaluation software (version 9.0).

### 4.3. Querying Interaction Databases and Large-Scale Datasets

Interactions of LOX and LOXLs were retrieved by manual curation of the literature and by querying manually curated interaction databases including the database we created, MatrixDB (http://matrixdb.univ-lyon1.fr/, [37,38,39,40]), and IntAct [42]. Both databases belong to the International Molecular Exchange (IMEx) consortium and follow its curation rules to capture interaction data [41,43]. Interaction partners of LOX and LOXLs were also identified in BioPlex 2.0 and 3.0 datasets [35,44] generated by affinity chromatography-mass spectrometry (AP-MS) profiling the entire human ORFeome collection in human embryonic kidney cells (HEK 293) and in a human colon cancer cell line (HCT 116). C-terminally FLAG-hemagglutinin-tagged baits from the human ORFeome v8.1 were expressed in HEK 293T or HCT 116 cells, the multi-protein complexes containing the baits and their prey were immunopurified and analyzed by liquid chromatography-mass spectrometry. AP-MS identifies interactions within multi-protein complexes and not binary interactions. Further details are available in [44], and on the BioPlex web site (https://bioplex.hms.harvard.edu/). 

### 4.4. Analyses of the Interaction Network of the LOX Family

Analyses were performed with several bioinformatic tools on proteins from the network identified by their gene names listed in Supplementary Table S1. Non-human partners of the LOX family were inferred to human orthologs. Only the canonical protein isoforms were considered for analyses, bioactive fragments were annotated using the terms associated with the gene encoding their parent proteins (e.g., COL18A1 for endostatin, the C-terminus part of collagen XVIII), and multimeric proteins (e.g., collagens, integrins, laminins and thrombospondins) were annotated with the terms annotating the genes encoding their constitutive polypeptides (e.g., LAMA1, LAMB1, and LAMC1 for laminin-111). 

The Functional Enrichment Analysis tool FunRich version 3.1.3 (http://www.funrich.org/, [73]) was used for functional enrichment and analysis of the LOX family interactome. The Biological Networks Gene Ontology (BiNGO) tool, which can be used directly on molecular interaction graphs [74], was used to determine which GO categories are statistically over-represented in the list of the LOX family partners. The human proteome was used as a reference, and the *p*-value was <0.01. Pathway over-representation was studied using Reactome knowledgebase version 74 (https://reactome.org, [75]). Pathways and network patterns related to cancer were determined using Reactome FIViz app available through Cytoscape. The class “Cancer-Related-Condition” (C8278) is defined as “A disorder either associated with an increased risk for malignant transformation (e.g., intraepithelial neoplasia, leukoplakia, dysplastic nevus, myelodysplastic syndrome) or that develops as a result of the presence of an existing malignant neoplasm (e.g., paraneoplastic syndrome)”.

### 4.5. Data Visualization

The networks were visualized using Cytotoscape 3.8.0 [76], and the Venn diagram was drawn with a freely available tool (http://bioinformatics.psb.ugent.be/webtools/Venn/). In order to color-code protein cellular location, their annotations from Gene Ontology (GO term “Cellular Component” [77,78]), manually checked with UniProtKB keywords, were retrieved from UniProtKB [79]. Matrisome and matrisome-associated annotations were retrieved from the Matrisome project (http://matrisome.org/) [80].

## 5. Conclusions 

The five members of the LOX family form a network comprised of 320 proteins, 5 GAGs, and 399 interactions, and share a single partner, the epidermal growth factor-like protein 7. LOX and LOXL2 have a high number of partners belonging to the matrisome, whereas LOXL1, LOXL3, and LOXL4 partners are mostly intracellular. The LOX family interactome is enriched in extracellular proteins, and participates in ECM organization (e.g., the formation of collagen fibrils and elastic fiber) and collagen degradation, “Chaperonin-mediated protein folding”, “Cellular response to external stimuli” pathway, “Cellular response to heat stress”, “Signal transduction” and “Organ development” including “blood vessel development”. The LOX family has twice more partners in tumor cells than in normal cells, which induces a pathway switch from ECM organization to chaperone activity, a decrease in extracellular and secreted proteins and in the transcriptional repressor CTCF. The LOX family interactome is thus significantly rewired in cancer cells.

Further binding assays will be performed to increase the coverage of the LOX interactome with proteins containing domains found in the known partners of LOX and LOXLs (e.g., the triple helix), and to determine the influence of mutations reported in cancer patients on the interaction network, and on the functional network in order to refine our understanding of the role of LOX and LOXLs in cancer. 

## Data Availability

The publicly available BioPlex datasets (https://bioplex.hms.harvard.edu/) were used in this study. The systematic atlas of matrisome data from the TGCA Pan-Cancer cohort used in this study was compiled by Izzi et al. (https://doi.org/10.1016/j.mbplus.2019.04.001).

## Supplementary Materials

The following are available online at https://www.mdpi.com/2072-6694/13/1/71/s1, Figure S1: LOXL2 binds to full-length α5β1 integrin in vitro. Figure S2: Proteins binding to LOXL2 and its N-terminal domains SRCR 1-2 and 1-4 by SPR binding assays. Figure S3: Partners of the SRCR 1-4 domain of LOXL2 identified by SPRi binding assays. Figure S4: Enrichment analysis of the GO term Biological Process for the LOX family interactome using BiNGO. Table S1: List of the interactions of the LOX family members. Table S2: Binding sites of LOXL2 partners identified by SPR imaging binding assays. Table S3: Cellular localization of the partners of the LOX family. Table S4: Enrichment analysis of the interactome of LOX family using BiNGO. Table S5: Enrichment analysis of the interactome of LOX family using FunRich. Table S6A-E: Enrichment analysis of the interactome of each member of the LOX family using FunRich. A: LOX, B: LOXL1, C: LOXL2, D: LOXL3, E: LOXL4. Table S7: The 100 most relevant Reactome pathways over-represented in the LOX family interactome, and in the partners of each member of the LOX family. Table S8A,B: Enrichment analysis of LOX family partners specific to HEK 293 cells (A) and HCT 116 cells (B) using FunRich. Table S9: The 20 most relevant Reactome pathways over-represented in the LOX family interactome of HEK and HCT 116 cells. Table S10: Matrisome proteins expressed in cancer from reference [53], list of the LOX family partners in mesothelioma (MESO) and ovarian serous cystadenocarcinoma (OV). Table S11A,B: Enrichment analysis of the subnetworks of the LOX family in mesothelioma (A) and ovarian serous cystadenocarcinoma (B) using FunRich. Table S12: Proteins used for in vitro binding assays with LOXL2 and its N-terminal domains.
