# Supplementary material for: The Interactome of Cancer-Related Lysyl Oxidase and Lysyl Oxidase-Like Proteins
Source: Cancers (Basel). 2020 Dec 29;13(1):71. doi: 10.3390/cancers13010071 (PMC7794802; doi:10.3390/cancers13010071)
Supplement: Supplementary file 1 [file cancers-13-00071-s001.zip › Supplementary_Material/Suppl_Figures_S1_S3.docx]

**Supplementary Figures**

The interactome of the LOX family, a cancer-related protein family.

Sylvain D. Vallet^1^, Coline Berthollier^1‡^, Romain Salza^1‡^, Laurent Muller^2^ and Sylvie Ricard-Blum^1,^*


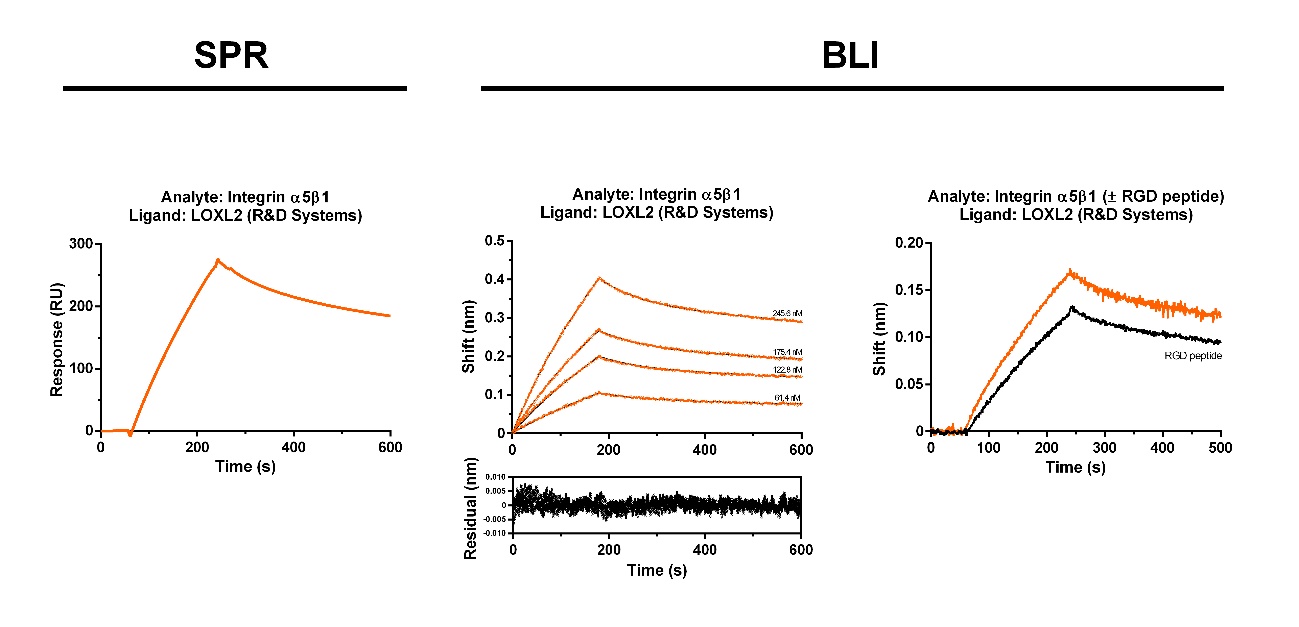


**Supplementary Figure S1.** **LOXL2 binds to full-length α5β1 integrin *in vitro*.** Direct interaction of LOXL2 covalently immobilized on a CM5 sensor chip to soluble α5β1 integrin identified by surface plasmon resonance (SPR) binding assay. Direct binding of LOXL2 covalently immobilized on a AR2G biosensor to soluble α5β1 integrin by bio-layer interferometry (BLI) binding assay. Orange: experimental sensorgrams, black: sensorgrams fitted to the heterogeneous ligand model (χ^2^ =0.0095). Inhibition experiments of α5β1 integrin binding to immobilized LOXL2 by preincubating the α5β1 integrin with the RGD peptide (50 µg/ml) as described in the Material and Methods section. The concentration of immobilized LOXL2 was 5 µg/ml and 25 µg/ml for SPR and BLI assays respectively.


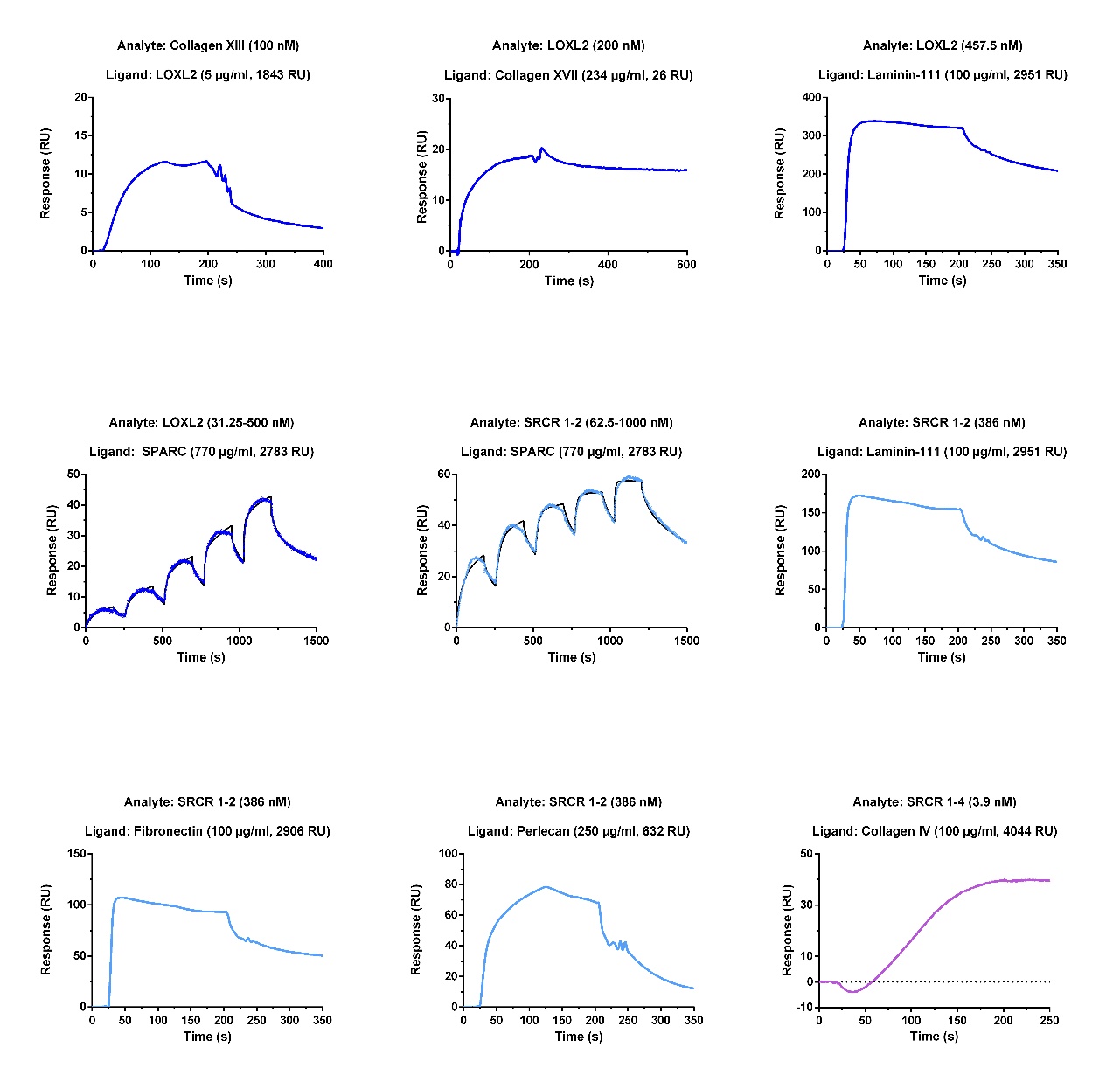


**Supplementary Figure S2**. **Partners of LOXL2 and of its N-terminal domains SRCR 1-2** (residues 58-302) **and SRCR 1-4** (residues 58-544) **identified by SPR binding assays.**


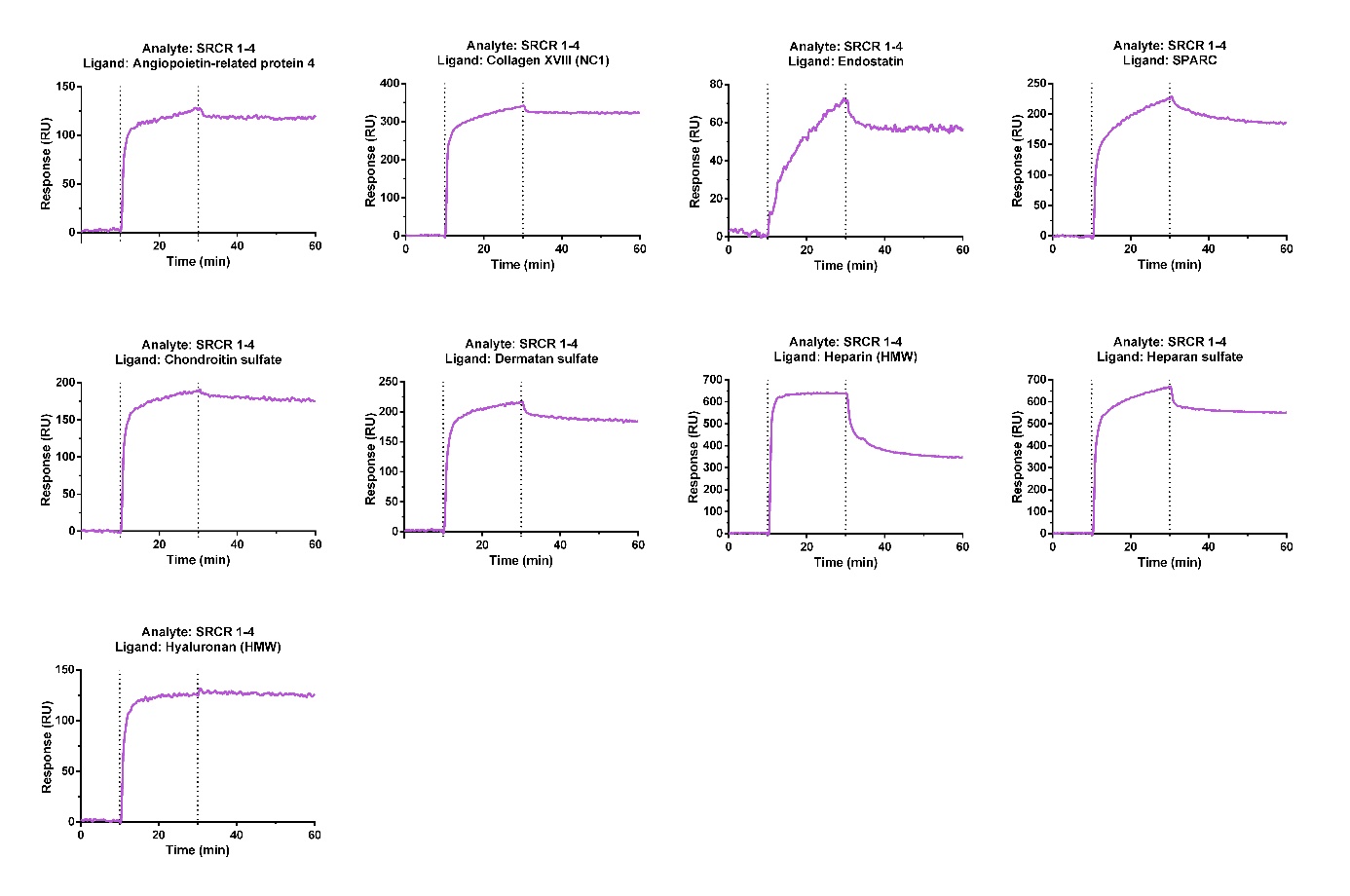


**Supplementary Figure S3. Partners of the SRCR 1-4 domain of LOXL2 identified by surface plasmon resonance imaging (SPRi) binding assays.** The N-terminal SRCR domain 1-4 was injected and recirculated over ECM protein and GAG arrays probed by SPR imaging. Non-specific binding to the array surface was subtracted from the raw signals obtained on protein and glycosaminoglycan spots to get specific binding. (HMW: high molecular weight, vertical dotted lines: start and end of the association phase).
