# Supplementary figures and images for: The Interactome of Cancer-Related Lysyl Oxidase and Lysyl Oxidase-Like Proteins
Source: Cancers (Basel). 2020 Dec 29;13(1):71. doi: 10.3390/cancers13010071 (PMC7794802; doi:10.3390/cancers13010071)

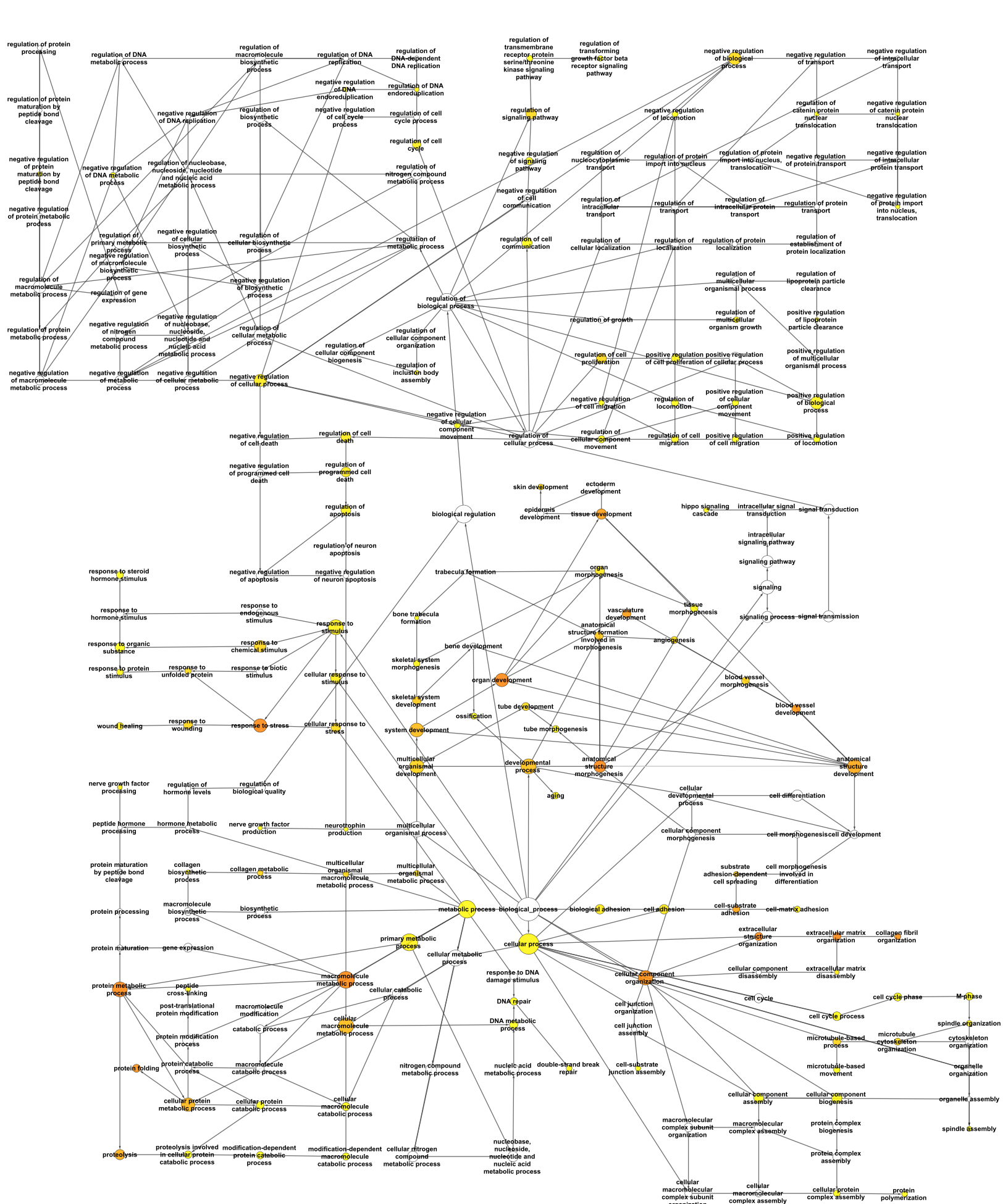

Supplement: Supplementary file 1 [file cancers-13-00071-s001.zip › Supplementary_Material/Suppl_Figure_S4.pdf]
